# *Nostoc calcicola* extract improved the antioxidative response of soybean to cowpea aphid

**DOI:** 10.1186/s40529-017-0211-9

**Published:** 2017-11-28

**Authors:** Van-Chung Mai, Ba-Hoanh Nguyen, Duc-Dien Nguyen, Le-Ai-Vinh Nguyen

**Affiliations:** 1 Department of Plant Physiology, School of Natural Sciences Education, Vinh University, str. Le Duan 182, Vinh, Nghe An Province Vietnam; 2 Department of Environmental Sciences, School of Chemo-Biology and Environment Technology, Vinh University, str. Le Duan 182, Vinh, Nghe An Province Vietnam

**Keywords:** *Aphis craccivora*, *Glycine max*, *Nostoc calcicola* HN9, Hydrogen peroxide, Superoxide anion radical, Catalase, Glutathione peroxidase, Superoxide dismutase

## Abstract

**Background:**

Soybean (*Glycine max* (L.) Merr. cv. “Nam Dan”) is one of the most valuable crops in agricultural production in Nghe An province (Vietnam). Our previous study revealed that extract of the cyanobacterium strain *Nostoc calcicola* HN9 expressed positive effect on growth and development, and raised soybean productivity (Tran et al. in Proceeding of Vietnam national conference of research on biology, Da Nang, 2016). We hypothesized that *N. calcicola* HN9 would improve the defense responses of *G. max* cv. “Nam Dan” to cowpea aphid (*Aphis craccivora* Koch)-a serious pest of leguminous crops.

**Results:**

Infestation of *A. craccivora* caused oxidative stress in leaves of *G. max* cv. “Nam Dan”. A strong generation of endogenous reactive oxygen species (ROS) such as superoxide anion radical (O_2_^·−^) and hydrogen peroxide (H_2_O_2_) resulted in the cellular damages in the aphid-infested leaves through high levels of injury percentage and lipid peroxidation. To protect from aphid attack themselves, soybean plants triggered the antioxidant defense systems, in which, enzymatic antioxidants such as superoxide dismutase (SOD, 1.15.1.1), catalase (CAT, 1.11.1.6) and GPx (EC 1.11.1.9) were strongly accumulated to reduce the toxic effects of ROS. Components of *N. calcicola* HN9 extract might strengthen the defensive capability of *G. max* cv. “Nam Dan” to cowpea aphid infestation via establishing the chemical constraints on oxidative stress. Under effect of cyanobacteria extract, generation of O_2_^·−^ and H_2_O_2_ was strictly limited, activities of SOD, CAT and GPx were remarkably accumulated in the aphid-infested leaves leading to a significant reduction of oxidative damages.

**Conclusions:**

*Nostoc calcicola* HN9 extract probably not only controlled the generation and effects of O_2_^·−^ and H_2_O_2_ but also augmented the accumulated activity of SOD, CAT and GPx in soybean leaves that allowed them to control oxidative stress, contributed to increase the resistance of *G. max* cv. “Nam Dan” to *A. craccivora*. The improvement of cyanobacteria extract on the antioxidative response of soybean “Nam Dan” to cowpea aphid can be a novel aspect to contribute to current knowledge regarding the soybean-aphid interaction.

## Background

Cyanobacteria (or blue-green algae) are the large group of prokaryotic, photosynthetic microorganisms, which are a rich source of potentially various bioactive compounds effecting on living organisms (Zulpa et al. 2003). The endogenous substances in cyanobacteria, e.g., vitamins, enzymes, carbohydrates, amino acids, have been known to induce crop growth and raise yields (Shariatmadari et al. 2013; Prasanna et al. 2013). Other natural products from cyanobacteria like carotenoids, phenolic compounds, etc., exhibit various biological effects such as cytotoxic, antibacterial and antifungal activities (Kulik 1995; Zulpa et al. 2003). Therefore, an inoculation with cyanobacteria not only promotes plants growth but also enhances their defense responses (Rana et al. 2012; Prasanna et al. 2013).

*Nostoc*, an unbranched filamentous cyanobacterium genus (Komárek 2010), has been regarded as good candidate for producing secondary substances that can effect on living organisms (Hirata et al. 2003). Treating *Solanum lycopersicum*, *Cucurbita maxima*, *Cucumis sativus* plants with *Nostoc calcicola* extracts revealed a significant positive effect in most physiological examined factors (Shariatmadari et al. 2011). The similar results were also confirmed by studies on other crops such as rice (Saadatnia and Riahi 2009), maize (Jäger et al. 2010). Several substances in *Nostoc* spp. expressed the antifungal, antibacterial, antiviral or cytotoxic activities on crops (Nowruzi et al. 2012). Nostocine A exhibited strong inhibitory activity to the root elongation of barnyard grass, high antifeedant activity to cotton ballworm, and acute toxicity to mice resulting in its classification as a dangerous poison (Hirata et al. 2003). Extract of *Nostoc* sp. induced oxidative stress by accumulating reactive oxygen species (ROS), causing massive cell death in *Mimosa pigra* (Sukkhaeng et al. 2015). Contrary, an in vitro study of Li and co-workers (2007) demonstrated that *Nostoc* species have their ability to scavenge ROS and inhibit lipid peroxidation.

Reactive oxygen species such as superoxide anion radical (O_2_^·−^), hydrogen peroxide (H_2_O_2_) are produced as a normal product of plant cellular metabolism. Despite their destructive activity, they are well-described second messengers in a variety of cellular processes, including conferment of tolerance to various stresses. Whether ROS would serve as signaling molecules or could cause oxidative damage to the tissues depends on the delicate equilibrium between ROS productions, and their scavenging (Sharma et al. 2012). In plants tissues affected by aphid attack, O_2_^·−^ and H_2_O_2_ are often enhanced in apoplastic as well as in symplastic regions, besides their main concentration in exocellular matrix, peroxisomes/mitochondria and plasma membrane (Smith and Boyko 2007; Maffei et al. 2007). High level of those ROS can exert toxic effects, and their uncontrolled productions can damage cellular components and associated loss of function leads to cell death (Ahmad et al. 2008). Plants have the efficient complex antioxidant defense systems to avoid the toxic effects of ROS, including enzymatic antioxidants such as superoxide dismutase (SOD), catalase (CAT), peroxidases (POX) and others (Maffei et al. 2007; Ahmad et al. 2008). Functions of those enzymatic antioxidants in plant defense responses have been reported in several plant-aphid interactions. Enhanced activity of SOD and CAT was suggested as one of the most essential elements of defense responses in pea seedling to oxidative stress caused by infestation of pea aphid (Mai et al. 2013). Accumulation of POX was strongly enhanced in wheat after feeding of the Russian wheat aphid (Moloi and van der Westhuizen 2006; Ni et al. 2001), in response of soybean to cowpea aphid (Mai et al. 2016). In cabbage plants, activities of polyphenol peroxidase and polyphenol oxidase were stimulated by cabbage aphid infestation (Khattab 2007). However, up to date, lack information has been mentioned about effects of cyanobacteria on plant antioxidant defense systems against aphid impacts.

Soybean (*Glycine max* (L.) Merr. cv. “Nam Dan”) is an important crop in the agricultural production in Vietnam for its edible seed as the foremost material of vegetative protein and oil used in the food processing. During its life, soybean plant has to face to attack from cowpea aphid (*Aphis craccivora* Koch)—one of the most destructive aphid species of leguminous crops. Our previous study (Tran et al. 2016) revealed that inoculation extract of the cyanobacterium strain *N. calcicola* HN9 presented the positive effect on growth, development and productivity of soybean “Nam Dan”. The aim of present work was to investigate effects of *N. calcicola* HN9 extract on the antioxidative responses of *G. max* cv. “Nam Dan” when soybean plants were cultured in Hoagland medium in phytotron, infested by different density of cowpea aphid in different time intervals. Cytochemical localization and the alteration in contents of O_2_^·−^, H_2_O_2_ as well as changes in activity of antioxidant enzymes such as SOD (EC 1.15.1.1), CAT (EC 1.11.1.6), and GPx (EC 1.11.1.19) in soybean leaves were carefully assessed. As a secondary objective, degree of cellular oxidative damages was investigated on measurement of the electrolyte leakage and levels of thiobarbituric acid reactive substances (TBARS)-a final product of lipid peroxidation. It is worth noting that the aforementioned aspects will provide the additionally convinced evidences to clarify whether *G. max* cv. “Nam Dan” is an aphid resistant cultivar of soybean.

## Methods

### Cyanobacteria culture and extraction

The cyanobacterium strain, *N. calcicola* HN9 (voucher specimen is deposited at herbarium of the Phycology lab, Vinh University), was collected from the rice field in Hung Nguyen district (Nghe An province, Vietnam) and isolated by the streak plate method. *Nostoc* cells were cultured in BG11 medium, pH 6.5 at ambient temperature (25 ± 2 °C), light intensity of 110–130 μM photons m^−2^ s^−1^, light period of 14 light/10 dark (Rippka et al. 1979).

The cyanobacteria biomass in the cultured day of 23th (in the stationary phase) was harvested, centrifuged at 6000×*g* for 15 min, and subsequently dried at 55 °C for 72 h. The dried cells were ground to powder, and extracted with 80% methanol for 24 h at ambient temperature. Supernatant was collected after centrifuging by 10,000×*g* for 15 min, then evaporated to obtain a crude brown gum. This gum was dissolved in the deionized water to the concentration of 0.03%, which expressed the positive effect to growth of soybean “Nam Dan” (Tran et al. 2016).

### Plant and inoculation experiment

Experiments were performed on soybean (*Glycine max* (L.) Merr. cv. “Nam Dan”) plants whose seeds were exclusively supplied by Nghe An seed center (Vietnam). Seeds were surface-sterilized by HgCl_2_ 0.01% for 10 min and then were imbibed in the incubator at 23 ± 1 °C for 48 h. Germinating seeds were cultured in 10 cm-diameter plastic pots (one plant per pot) containing Hoagland medium placed in phytotron with temperature of 23 ± 1 °C, relative humidity of 70–75%, light intensity of 110–130 μM m^−2^ s^−1^ and light period 14 light/10 dark hours.

As growing in the V3 stage (first two trifoliate leaves fully developed, third trifoliate leaf unrolled), soybean plants were divided into two equal groups. The first one included soybean plants that were sprayed on the surface of leaves by 1 mL solution *N. calcicola* HN9 extract, concentration 0.03%, per plant. The second one was soybean without cyanobacteria inoculation.

### Aphid and infestation experiment

The aphid species used to treat soybean “Nam Dan” is cowpea aphid (*Aphis craccivora* Koch), which is obtained from Department of Applied Entomology (Vietnam Academy of Science and Technology). The virus free individuals were cultured on host, *G. max*, in phytotron in temperature of 23 ± 1 °C, relative humidity of 70–75%, light intensity of 110–130 μM m^−2^ s^−1^ and light period of 14 light/10 dark hours (Mai et al. 2016).

After spraying of *N. calcicola* HN9 extract 24 h, all soybean plants in two mentioned groups were used for setting up experiments of infestation. Each soybean plant was separately treated by 10, 20 or 30 wingless adults of *A. craccivora*. The similarly wingless adults of cowpea aphid were carefully selected and transferred to soybean leaves with a fine paintbrush. The newly produced nymphs and winged individuals were monitored daily to prevent density-dependent effects on aphid performance; therefore, number of wingless adults of *A. craccivora* in experiments was constant. Control was soybean plants without aphid infestation. The control and aphid-infested variants were separately put in glass boxes (50 cm × 50 cm × 50 cm) covered by nylon gauze; all were placed in the phytotron with the environmental factors such as temperature, related humidity, light intensity and light period controlled.

### Material for analysis

Mature leaves of soybean plants in all variants were carefully collected at 0, 6, 12, 24, 48, 72 and 96 h post-infestation (hpi) of cowpea aphid. Aphid individuals all were removed; leaves were weighed, frozen in nitrogen liquid and kept at − 20 °C for subsequent analyses of lipid peroxidation and activity of antioxidant enzymes. Content and cytochemical localization of superoxide anion radical (O_2_^·−^), hydrogen peroxide (H_2_O_2_) and injury percentage were determined in fresh materials.

### Determination of superoxide anion radical content

Content of superoxide anion radical (O_2_^·−^) was determined following the nitro blue tetrazolium (NBT) assay (Doke 1983). Soybean leaves (0.30 g fresh weight-FW) were incubated in 3 mL of 10 mM phosphate buffer (pH 7.8) containing 0.05% NBT and 10 mM NaN_3_ at ambient temperature for 1 h. After incubation, the reacted solution was heated at 85 °C for 15 min, then rapidly cooled. Measurements were carried out in the UV–Vis CARY 60 spectrophotometer (Agilent, USA) connected with a computer; the spectral data were analyzed by using the UV-Win 5.0 application software. Content of O_2_^·−^ was expressed as absorbance at 580 nm per 1 g fresh materials (A_580_ g^−1^ FW).

### Detection of superoxide anion radical localization

The cytochemical localization of O_2_^·−^ was detected following method using dihydroethidium (DHE), the specific fluorescent dye, described by Morkunas and Bednarski (2008) with minor modification. The fresh soybean leaves were submerged in 50 M DHE in 5 mM DMSO after immersing 12 h in darkness at ambient temperature. After rinsing with the 100 M CaCl_2_ solution pH 5.0, the DHE dying leaves were observed by using a Zeiss Axiovert 200 M fluorescence microscope (model LSM 510, filter set no. 9, excitation 450–490 nm, emission 520 nm or more), magnified 5× and photographed by a digital camera (AxioCam, Zeiss). An argon laser with excitation at 488 nm and emission at 565–615 nm was used. Microscope, laser and photomultiplier settings were held stably during experiments to obtain comparable data. Images were analyzed by the LSM Image Browser software, version 4.2.

### Determination of hydrogen peroxide concentration

Concentration of endogenous hydrogen peroxide (H_2_O_2_) was determined following the spectrophotometric method (Becana et al. 1986) that was modified by Mai et al. (2013). 0.50 g fresh material were homogenized at 4 °C with 3 mL of 5% trichloroacetic acid (TCA) and 0.10 g activated charcoal. The homogenate was centrifuged at 12,000×*g* for 30 min at 4 °C to select supernatant using as extract. The reagent was prepared of 0.6 mM 4-(-2 pyridylazo)resorcinol, 0.6 mM of potassium-titanium oxalate in 1:1 proportion. Total 3 mL the reaction mixture contained extract, 100 mM phosphate buffer (pH 8.4) and reagent. The decrease of absorbance was measured at wavelength of 508 nm in the UV–Vis CARY 60 spectrophotometer (Agilent, USA) system. The spectral data were analyzed by using the UV-Win 5.0 application software. Amount of hydrogen peroxide in soybean leaves was expressed as M H_2_O_2_ g^−1^ FW.

### Detection of hydrogen peroxide localization

Cytochemical localization of endogenous H_2_O_2_ generated in soybean leaves was detected by staining with specific fluorescence, the 2′,7′-dichlorofluorescein diacetate (DCFH-DA) following methods of Małecka et al. (2009) with minor modification. The fresh soybean leaves were submerged in 4 M DCFH-DA dissolved in 50 mM potassium phosphate buffer (pH 7.5) for 12 h. Leaves were washed twice with the loading buffer and then were observed with the Zeiss LSM 510 confocal microscopy system. Fluorescence was excited using 488 nm of an argon laser with emission at 500–550 nm. All images were obtained at the same depth, and were analyzed by the LSM Image Browser software, version 4.2.

### Electrolyte leakage

Electrolyte leakage was the conductometrical method used for assessing the injury percentage of cell membrane (Bajji et al. 2002). Five fresh, complete leaves of each sample were washed in deionized water for a few seconds, embedded in test-tubes containing 20 mL of deionized water and took the first conductometric measurement (EC_i_); then incubated on a shaking platform at ambient temperature for 3 h and measured again (EC_f_) by a conductivity meter (Orion Star A212, Thermo Scientific, USA). After autoclaving the solution at 100 °C for 20 min to rupture completely the membranes allowing ion leakage of the entire cell contents and cooling to ambient temperature, the third measurement was done (EC_t_). The membrane damage of soybean leaves’ cells was evaluated as the injury percentage compared with control and was calculated as ${\text{I}}_{\text{d}} = \left[ {\left( {{\text{R}}_{\text{s}} - {\text{R}}_{\text{c}} } \right)/\left( {1 - {\text{R}}_{\text{c}} } \right)} \right] \times 100 \, \left( \% \right);$ where R_s_ and R_c_ represent (EC_f_ − EC_i_)/(EC_t_ − EC_i_) for aphid and/or cyanobacteria treated tissues and control, respectively.

### Lipid peroxidation

The thiobarbituric acid reactive substances (TBARS) assay is the most generally used test in the appreciation of lipid peroxidation (Heath and Packer 1968). 0.50 g of frozen soybean leaves was homogenized in 3 mL of 0.5% thiobarbituric acid (TBA) in 20% TCA and then centrifuged at 12,000×*g* for 20 min at 4 °C to select the extract. The reaction mixture [extract, phosphate buffer (pH 7.0) and reagent (0.5% TBA in 20% TCA, w/v)] were incubated at 95 °C for 30 min and then quickly cooled in an ice bath. After that, the mixtures were centrifuged at 10,000×*g* for 10 min. The specific absorbance at 532 nm and the non-specific absorbance at 600 nm of the clear supernatant were measured by using the UV–Vis CARY 60 spectrophotometer. Level of lipid peroxidation was calculated and expressed as μM TBARS g^−1^ FW.

### Enzyme assay

Briefly, 0.50 g frozen soybean leaves were homogenized at 4 °C in 5.0 mL of 50 mM phosphate buffer (pH 7.0) containing 1.0 mM EDTA, 2% NaCl and 1% polyvinyl pyrrolidone and centrifuged at 15,000×*g* for 15 min to select supernatant as the enzymatic extract.

Activity of SOD (EC 1.15.1.1) was spectrophotometrically assayed by measuring its ability in inhibition the photochemical reduction of NBT to a blue solution (Beauchamp and Fridovich 1971). The reacted mixture contained 50 mM phosphate buffer (pH 7.8), 13 mM methionine, 75 mM NBT, 0.1 mM EDTA, enzymatic extract and 2 mM riboflavin. The reaction was started by switching on a 30 W fluorescent lamp placed 30 cm above test-tubes and proceeded for 15 min. Samples without the enzymatic extract were selected so that the absorption difference between blank and examined tests was about 50%. Measurements were done by using the UV–Vis CARY 60 spectrophotometer. Amount of enzyme that caused the inhibition of NBT reduction by 50% was taken as a unit of SOD activity.

Activity of CAT (EC 1.11.1.6) was determined by measuring hydrogen peroxide removal (Dhindsa et al. 1981). The reacted mixture contained 100 mM phosphate buffer (pH 7.0), 3% H_2_O_2_ and enzymatic extract. Absorbance was assessed by measuring at 240 nm against a calibration curve using the UV–Vis CARY 60 spectrophotometer.

Activity of GPx (EC 1.11.1.19) was assayed according to the procedure of Flohé and Günzler (1984) with minor modifications. The reaction mixture consisting of 0.5 mL of each: 0.4 M sodium phosphate buffer (pH 7.0), 10 mM sodium azide, 4 mM reduced glutathione, 5 mM H_2_O_2_, and enzyme extract was incubated at 0, 30, 60, 90 s respectively. The reaction was terminated with 0.5 mL of 10% TCA and centrifuged; after that 2 mL of the supernatant was added to 3 mL of phosphate buffer and 1 mL of DTNB reagent (0.04% DTNB in 1% sodium citrate). Absorbance was measured at a wavelength of 412 nm in the UV–Vis CARY 60 spectrophotometer.

Activity of SOD, CAT and GPx was expressed as nanokatal per 1 mg of protein (nkat mg^−1^ protein). The protein concentration in the samples was determined according to Bradford (1976) method by using bovine serum albumin as a standard.

### Statistical analysis

Analyses all were performed at least in three independent experiments. Analysis of variance (ANOVA) was applied to verify whether means from independent experiments within each given variant were significant at level P < 0.05. Data shown in the figures are means and standard errors (s.e.), using asterisks to present the significant differences.

## Results

### Generation of ROS in leaves of soybean “Nam Dan”

Generally, a strongly release the reactive oxygen species (ROS) productions such as superoxide anion radical (O_2_^·−^) and hydrogen peroxide (H_2_O_2_) was observed in aphid-infested leaves of soybean (*Glycine max* cv. “Nam Dan”) in stage V3.

**Fig. 1:**
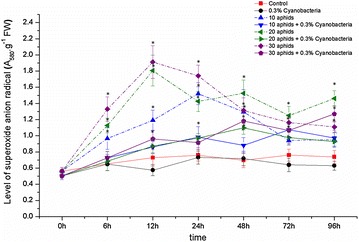
Generation of superoxide anion radical (O_2_^·−^) in leaves of *Glycine max* cv. “Nam Dan” under infestation of *Aphis craccivora* and inoculation of *Nostoc calcicola* HN9 extract. Values represent means and s.e. from three independent experiments. (*) In figure shown the significant difference between the experimental variants and control at level P < 0.05

Without inoculating *N. calcicola* HN9 extract, superoxide anion radical in the aphid-infested leaves was remarkably released and reached to the highest content at 12 h post-infestation (hpi) of 10, 20, or 30 aphids, having values of 1.64-, 2.48-, and 2.63-fold higher than at the beginning, respectively. This ROS molecule then slightly reduced to lower levels until 96 hpi. Whereas, this free radical in control plants maintained at low level throughout the experiment (Fig. 1; the dash lines). ANOVA results showed the significant difference in levels of O_2_^·−^ in aphid-infested variants and control within 6–48 hpi.

Contrary, a small amount of O_2_^·−^ was generated in leaves inoculated with *N. calcicola* HN9 extract, and content of this free radical was slightly increased from the beginning up to 96 hpi (Fig. 1; the solid lines). In most of investigated time points, content of O_2_^·−^ in soybean treated by *N. calcicola* HN9 extract was in lower than that in plant without cyanobacteria inoculating.

**Fig. 2:**
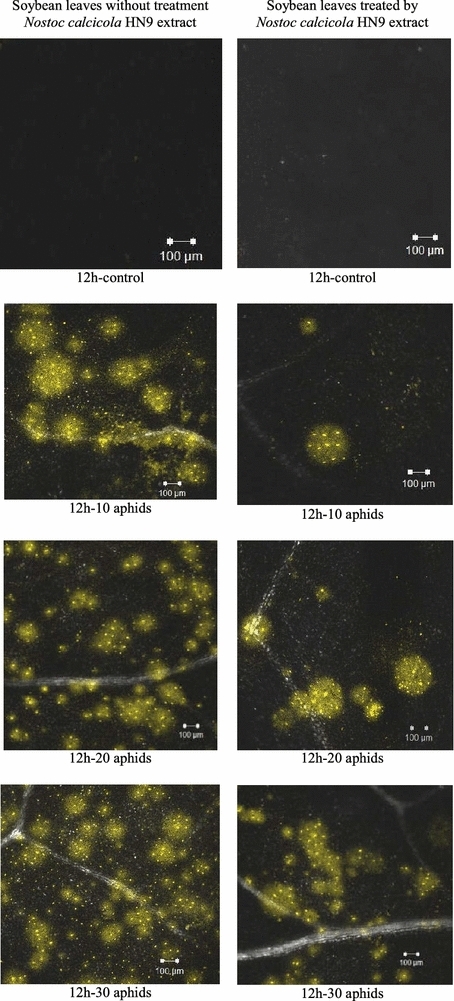
Cytochemical localization of superoxide anion generation (O_2_^·−^) in leaves of *Glycine max* cv. “Nam Dan” under infestation of *Aphis craccivora* and inoculation of *Nostoc calcicola* HN9 extract at 12 hpi. Yellow fluorescence came from DHE (dihydroethidium), which is observed by a Zeiss LSM 510 confocal microscope, with excitation at 488 nm, emission at 565–615 nm, objective magnification of 5×, scale bar 100 µm

The applying confocal microscopy results revealed the relative release of O_2_^·−^ in soybean leaves via DHE-fluorescence staining in epidermis cells surrounding the aphid feeding sites. DHE can passively cross the cells membrane, and is oxidized by O_2_^·−^ to ethidium bromide that is detected by using the confocal laser scanning microscope. The fluorescence is proportional to the intracellular O_2_^·−^ level. Figure 2 presented an example about the presence of O_2_^·−^ at 12 hpi, in which time a burst of O_2_^·−^ in aphid-infested/non cyanobacteria inoculated leaves was recorded by the spectrophotometric method. Cytochemical localization of O_2_^·−^ presented that, a stronger intensity and larger surface area of the DHE-derived fluorescence appeared in leaves only infested by *A. craccivora*; while a small fluorescent stain was observed in the additionally inoculated cyanobacteria leaves. The fluorescent intensity also provided an important evidence that the release of O_2_^·−^ was limited in soybean leaves inoculated by *N. calcicola* HN9 extract.

**Fig. 3:**
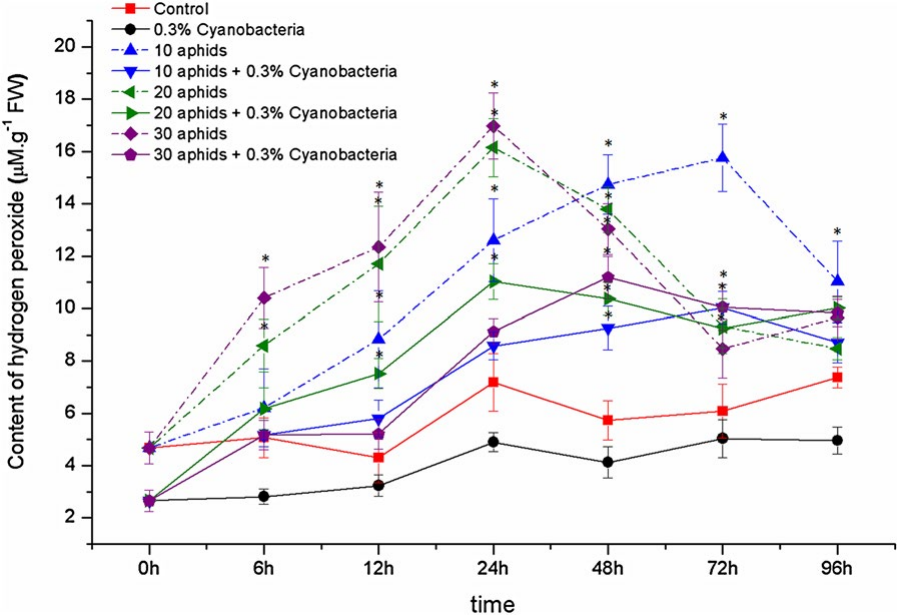
Generation of hydrogen peroxide (H_2_O_2_) in leaves of *Glycine max* cv. “Nam Dan” under infestation of *Aphis craccivora* and inoculation of *Nostoc calcicola* HN9 extract. Values represent means and s.e. from three independent experiments. (*) In figure shown the significant difference between the experimental variants and control at level P < 0.05

A strong generation and continuous increase of H_2_O_2_ in aphid-infested leaves was observed in soybean plants without cyanobacteria inoculating within 0–48 hpi (Fig. 3; the dash lines). The maximum levels of this ROS production were obtained at 24 hpi in 20- and 30-aphid infested leaves, whereas content of H_2_O_2_ in 10-aphid infested variant was continuously increased until 72 hpi. Infestation of 30 cowpea aphids per plant caused to release the highest amount of H_2_O_2_, obtaining 16.97 mM g^−1^ FW, having by 2.36- and 3.63-fold higher than in control and from beginning, respectively. The most remarkable difference was recorded between content of H_2_O_2_ in aphid-infested and control plants within 6–48 hpi. Similar to O_2_^·−^, an accumulation of H_2_O_2_ in leaves inoculated with *N. calcicola* HN9 was much lower than that observed in soybean without inoculating cyanobacteria (Fig. 3; the solid lines).

**Fig. 4:**
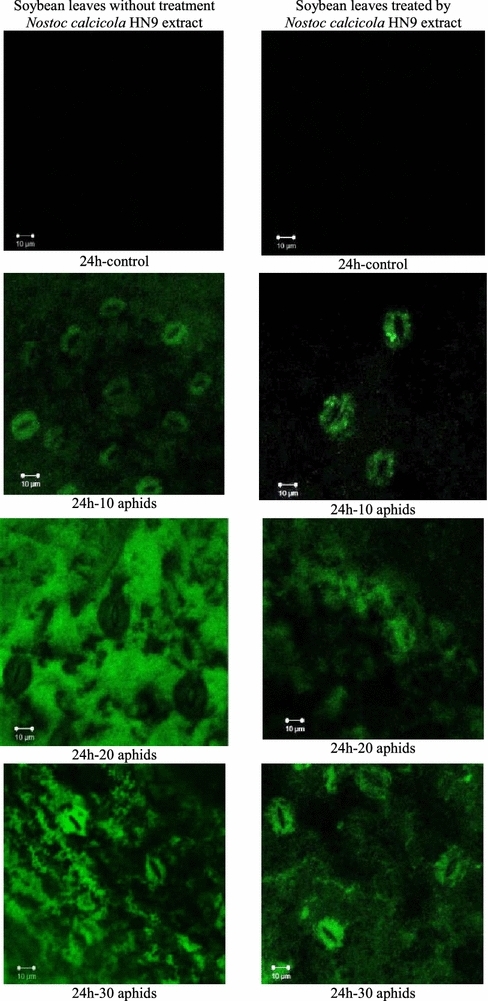
Cytochemical localization of hydrogen peroxide (H_2_O_2_) in leaves of *Glycine max* cv. “Nam Dan” under infestation of *Aphis craccivora* and inoculation of *Nostoc calcicola* HN9 extract at 24 hpi. Green fluorescence came from DCFH-DA (dichlorodihydro-fluorescein diacetate), which is observed by a Zeiss LSM 510 confocal microscope, with excitation at 488 nm, emission at 500–550 nm, objective magnification of 63×, scale bar 10 µm

In addition, observations of the relative generation H_2_O_2_ in soybean leaves using confocal microscopy revealed similar trends to the spectrophotometric results. After staining with 2′,7′-dichlorofluorescein diacetate (DCFH-DA) there was observed DCFH-DA-derived fluorescence in soybean leaves. DCFH-DA is a nonpolar dye, converted into the polar derivative DCFH that are non-fluorescent but switched to highly fluorescent DCF when oxidized by intracellular H_2_O_2_. DCF can be observed as green color under the fluorescent microscope and intensity of fluorescence is proportional to the endogenous H_2_O_2_ level. A high intensity of fluorescence was observed in the aphid-infested leaves and covered a large uniform area of tissue, whereas no or small trace of fluorescent staining was detected in control (e.g., at 24 hpi, Fig. 4). Intensity of green color in *N. calcicola* HN9-inoculated leaves was remarkably lower in comparing with that recorded in soybean without cyanobacteria treatment.

### Cell membrane injury

The membrane injury degrees in *G. max* cells were estimated through assessment of electrolyte leakage and lipid peroxidation.

**Fig. 5:**
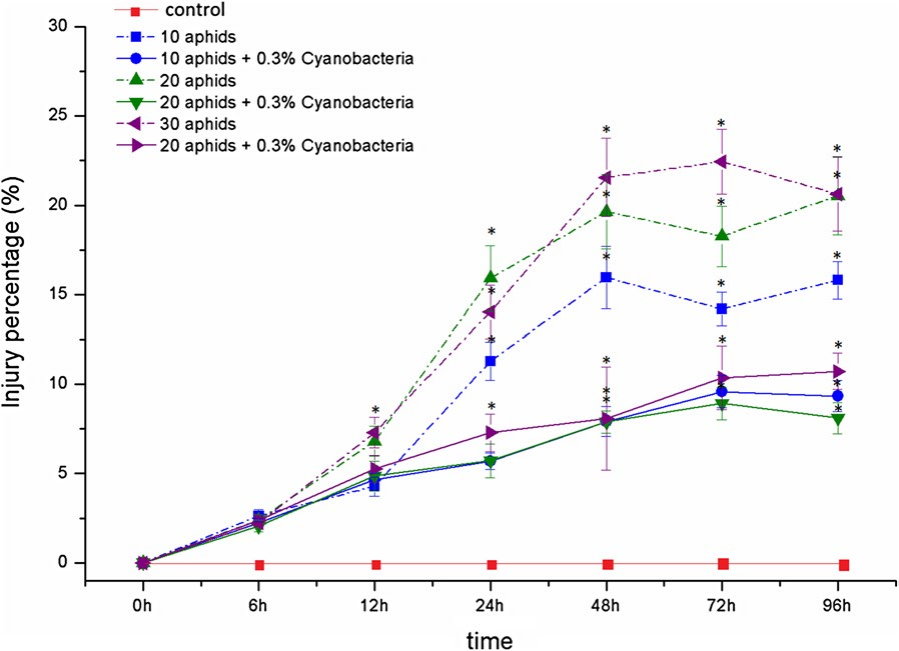
The percentage of injury (in comparing with control) in *Glycine max* cv. “Nam Dan” leaves under infestation of *Aphis craccivora* and inoculation of *Nostoc calcicola* HN9 extract. Values represent means and s.e. from three independent experiments. (*) In figure shown the significant difference between the experimental variants and control at level P < 0.05

The electrolyte leakage of soybean leaves’ cells was evaluated as the injury percentage compared with control. Without inoculating with *N. calcicola* HN9 extract, the injury percentage in all aphid-infested leaves increased from 0 to 48 hpi and maintained in high levels until 96 hpi (Fig. 5; the dash lines). The highest degree of cellular injury was recorded at 72 hpi in leaves infested by 30 aphids, having 22.45% higher than in control.

Inoculation of *N. calcicola* HN9 extract seemed to reduce injury rate in the aphid-infested leaves. The injury percentage in soybean inoculated with *N. calcicola* HN9 extract was always significant lower than that observed in plants without treating cyanobacteria. The maximum degree of injury recorded in 30-aphid infested leaves at 96 hpi was only 9.72% (Fig. 5; the solid lines).

**Fig. 6:**
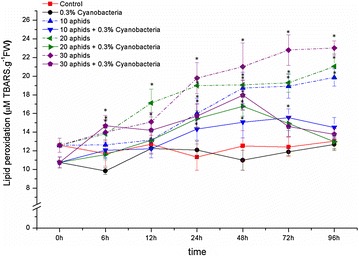
Lipid peroxidation in leaf tissues of *Glycine max* cv. “Nam Dan” leaves under infestation of *Aphis craccivora* and inoculation of *Nostoc calcicola* HN9 extract. Values represent means and s.e. from three independent experiments. (*) In figure shown the significant difference between the experimental variants and control at level P < 0.05

Infestation of *A. craccivora* induced the lipid peroxidation process in *G. max* cv. “Nam Dan” leaves (Fig. 6; the dash lines). A small but progressive increase in content of TBARS, the final products of lipid peroxidation, reached the highest level at 96 hpi, e.g., 19.87, 21.04 and 23.02 M TBARS g^−1^ FW under effect from 10, 20 and 30 aphids, respectively. ANOVA results showed that, content of TBARS in aphid-infested leaves was significantly higher than in control, and an appreciable proportion between levels of TBARS and the infestation intensity of cowpea aphid was exposed since 24 hpi.

Similar to effect on injury percentage of cellular membrane, *N. calcicola* HN9 extract might control lipid peroxidation. This process in plants inoculated with cyanobacteria extract was minor changed during the investigated time; TBARS content was mostly smaller than that observed in soybean without inoculating cyanobacteria (Fig. 6; the solid lines). The maximum content of TBARS obtained in 30-aphid infested/*N. calcicola* HN9 inoculated leaves at 48 hpi was 17.96 M TBARS g^−1^ FW that was only 78.02% in comparing with that in 30-aphid infested leaves.

### Activity of enzymatic antioxidants

Analyses of spectrophotometric assays showed that infestation of *A. craccivora* and inoculation *N. calcicola* HN9 extract differently induced activity of enzymatic antioxidants such as SOD, CAT and GPx in leaves of *G. max* cv. “Nam Dan”.

**Fig. 7:**
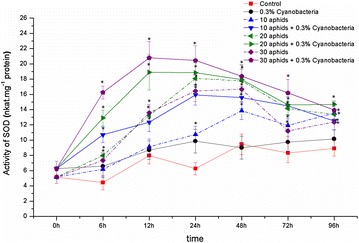
Activity of superoxide dismutase (SOD) in *Glycine max* cv. “Nam Dan” leaves under infestation of *Aphis craccivora* and inoculation of *Nostoc calcicola* HN9 extract. Values represent means and s.e. from three independent experiments. (*) In figure shown the significant difference between the experimental variants and control at level P < 0.05

Activity of SOD in soybean leaves infested by different densities of cowpea aphid increased in the first 24 h of infestation. The highest value of SOD activity recorded in 20 aphid-infested leaves at 24 hpi was 18.08 nkat mg^−1^ protein, which was 288.9 and 351.3% higher than SOD activity in control and at the beginning, respectively. After reaching the peak within 24–48 hpi, activity of this enzyme was strongly reduced to low levels but always higher than in control (Fig. 7; the dash lines). ANOVA analyses confirmed the significant differences between the SOD activities in the aphid-infested and control plants during experimental time. However, no appreciable proportion between the infestation intensity and the enhanced activity of SOD was recorded.

Inoculation soybean with *N. calcicola* HN9 extract sooner accumulated action of SOD in the aphid-infested leaves. Activity of this enzyme was strongly increased to peak within 12–24 hpi, and maintained in high level up to 96 hpi. There was an appreciable proportion between the accumulated activity of SOD and intensity of aphid infestation within 6–72 hpi. The highest activity of SOD enhanced by infestation of 30 aphid individuals at 12 hpi was 20.79 nkat mg^−1^ protein, having by 2.61- and 3.32-fold higher than that in control and at the beginning, respectively (Fig. 7; the solid lines). ANOVA analyses confirmed that SOD activities in the aphid-infested/cyanobacteria inoculated soybean leaves were significant higher than that in control plants within 6–96 hpi.

**Fig. 8:**
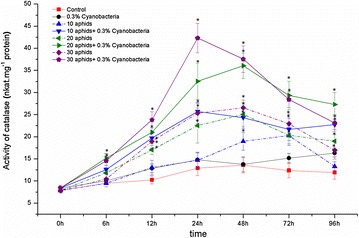
Activity of catalase (CAT) in *Glycine max* cv. “Nam Dan” leaves under infestation of *Aphis craccivora* and inoculation of *Nostoc calcicola* HN9 extract. Values represent means and s.e. from three independent experiments. (*) In figure shown the significant difference between the experimental variants and control at level P < 0.05

Similar to SOD, enzyme CAT in leaves of soybean “Nam Dan” was also induced after cowpea aphid infestation. An accumulated activity of CAT was observed from the beginning, reached to peak at 48 hpi. The highest activity of CAT obtained in 30-aphid infested leaves were 26.54 nkat mg^−1^ protein, having by 195.49% higher than in the control (Fig. 8; the dash lines). ANOVA results revealed a significant difference between the activities of CAT in 20- and 30-aphid infested soybean plants and control within 12–72 hpi.

Under effect of *N. calcicola* HN9 extract, an explosive expression of CAT was observed in aphid-infested leaves. Activity of CAT was remarkably increased and reached to peak within 24–48 hpi. The highest activity of CAT in 30-aphid infested soybean was 42.31 nkat mg^−1^ protein at 24 hpi, having by 1.59-folder higher than the maximum activity of CAT in plants without inoculating cyanobacteria (26.54 nkat mg^−1^ protein). Interestingly, an appreciable proportion between activity of CAT and the infestation intensity of cowpea aphid was also recorded (Fig. 8; the solid lines).

**Fig. 9:**
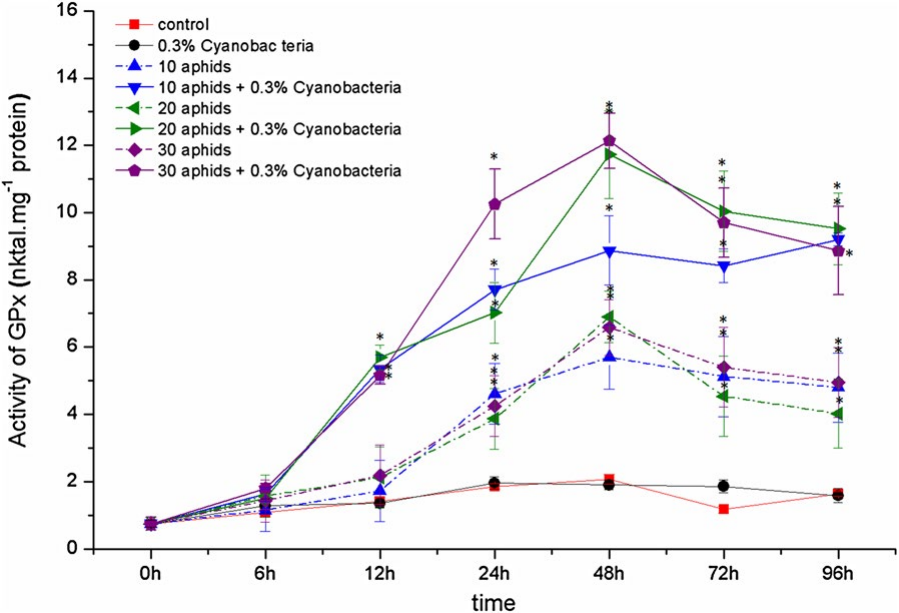
Activity of glutathione peroxidase (GPx) in *Glycine max* cv. “Nam Dan” leaves under infestation of *Aphis craccivora* and inoculation of *Nostoc calcicola* HN9 extract. Values represent means and s.e. from three independent experiments. (*) In figure shown the significant difference between the experimental variants and control at level P < 0.05

Cowpea aphid infestation also enhanced activity of GPx in leaves of soybean “Nam Dan”. Activity of this enzyme was accumulated since 12 hpi, increased to maximum at 48 hpi, and then slightly reduced to ending of experiments (Fig. 9; the dash lines). The highest activity of GPx obtained in 20 aphid-infested leaves at 48 hpi was 6.91 nkat mg^−1^ protein, having by 9.24- and 3.32-fold-higher than that at the beginning and control, respectively.

Inoculating soybean “Nam Dan” by *N. calcicola* HN9 extract seemed to improve action of GPx in plants infested by cowpea aphid. This antioxidant enzyme in the inoculated leaves was soon induced since 6 hpi and prolonged high activity until 96 hpi. Under effect of *N. calcicola* HN9 extract, the maximum activity of GPx recorded in 30-aphid infested plants at 48 hpi was 12.13 nkat mg^−1^ protein, having by 175.54% that observed in soybean without inoculating cyanobacteria. There was a significant difference between activity of GPx in the aphid-infested plants and control since 6 hpi, however, the relationship between the accumulated enzymatic activity and intensity of infestation was unappreciable (Fig. 9; the solid lines).

## Discussion

Feeding of insects, including aphids, has been known to induce generation of the endogenous ROS such as O_2_^·−^ and H_2_O_2_ in plants (Orozco-Cárdenas and Ryan 1999; Ni et al. 2001; Argandona et al. 2001; Zhu-Salzman et al. 2004; Moloi and van der Westhuizen 2006; Ślesak et al. 2007; Kuśnierczyk et al. 2008; Rangasamy et al. 2009; Liu et al. 2010; Radville et al. 2011; Morkunas et al. 2011; Mai et al. 2013, 2016). Similar to previous studies, infestation of *A. craccivora* accumulated a strong release of O_2_^·−^ and H_2_O_2_ in leaves of *G. max* cv. “Nam Dan” (Figs. 1, 3). The induced generation of O_2_^·−^ constitutes an important aspect of plant potential defense (Mai et al. 2013). Induction of O_2_^·−^ within a short time after stress mediated rapid local resistance mechanisms in plants against insect larvae, leading to cell death (Bown et al. 2002), might play an important function in protection against infection by opportunistic pathogens at the site of the larval footprint damage (Hall et al. 2004). A large amount of O_2_^·−^ in cell wall may limit the pathogen invasion that can be directly transmitted from aphid saliva during its penetration into plant tissues (Capinera 2001). Different from O_2_^·−^ functions, enhancement of H_2_O_2_ stimulates a cascade of many physiological and molecular responses (Maffei et al. 2007). H_2_O_2_ causes to release of Ca^2+^ flux into the cellular matrix, which is the control point for signal transduction processes or activates some members of the mitogen-activated protein kinase (MAPK) family-central for mediating cellular responses to stresses (Neill et al. 2002). H_2_O_2_ can tighten the cell wall by driving lignification as well as enabling the oxidative coupling of polysaccharide-bound phenolics, which strengthen barriers to aphid probing within plant tissues (Vreeburg and Fry 2005; Gapper and Dolan 2006), leading to prevent or minimize aphid attack (Argandona et al. 2001), reflecting a defensive role on protection plant tissues from wounding.

However, high levels of ROS can exert toxic effects and caused “oxidative stress” to plant cells. The uncontrolled productions of ROS can result in damages of cellular components such as proteins, lipids, and nucleic acids (Ahmad et al. 2008), often associated cell membrane injury leading to electrolyte leakage and lipid peroxidation. As aphid probing into plant tissues, components of sheath and watery saliva may establish the physical and chemical properties to cause the extensive cellular disruption. An increased electrolyte leakage is used to indicate loss of membrane integrity, and soybean cells membranes may be subject to changes under cowpea aphid infestation. Lipid peroxidation involves the formation of lipid radicals, the eventual destruction of membrane lipids, is also considered as the oxidative damage to cell structures that may lead to cell death (Dianzani and Barrera 2008). Under effects from different densities of *A. craccivora*, an extension of electrolyte leakage and a progressive increase in content of TBARS-the final products of lipid peroxidation were observed in leaves of *G. max* cv. “Nam Dan” (Figs. 5, 6; the dash lines). That was certain symptoms of cellular damage resulting from oxidative stress caused by cowpea aphid infestation.

Regarding effects of inoculation cyanobacteria to crops, several previous reports showed that cyanobacterial materials could induce productions of oxidants. *Nostoc* sp. extract caused a burst of O_2_^·−^ and H_2_O_2_ generation leading to the cellular damages in root of *Mimosa pigra* (Sukkhaeng et al. 2015). Lipid peroxidation in *M. pigra* and *Vallisneria natans* plants also significantly increased after treatment of cyanobacterial extract (Jiang et al. 2011; Sukkhaeng et al. 2015). The cyanobacterial bloom products caused an oxidative damage in *Medicago sativa* seedlings (Pflugmacher et al. 2006). Furthermore, some bioactive substances from cyanobacteria extract can be toxic to plant tissues, even result in cell death. For example, Nostocine A produced by *N. spongiaeforme* TISTR 8169 exhibited an inhibitory activity on root growth of barnyard grass (Hirata et al. 2003). Microcystin-RR from *Microcystis aeruginosa* decreased cell viability of tobacco after inoculation (Yin et al. 2005). Different from the above results, *Nostoc* species have several antioxidant properties, due to their ability to scavenge free radicals such as O_2_^·−^ and OH^·^ and inhibit lipid peroxidation (Li et al. 2007). In this light, our study also revealed that, inoculation of *N. calcicola* HN9 extract depressed generation of O_2_^·−^ and H_2_O_2_; levels of those ROS remarkably decreased in leaves of *G. max* cv. “Nam Dan” infested by *A. craccivora*. Extract of this cyanobacterium strain may exhibit potential adaptation strategies against oxidative stress in soybean “Nam Dan”. The extract could activate plant antioxidant defense system that comprises various enzymatic antioxidants that mainly caused a reduction of ROS content (Bharanidharan et al. 2013; Guedes et al. 2013). Moreover, cyanobacteria extract also contains phenolic acids, flavonoids and other non-enzymatic antioxidants that improve plant adaptive responses to oxidative stress (Honga et al. 2008; Krishnaraj et al. 2012). Phenolic acids and flavonoids are able to donate hydrogen atom to the free radical thus stopping the propagation chain reaction during lipid peroxidation process (Abd El-Baky et al. 2009). It is known that prevention of the chain initiation step by scavenging ROS products is considered to be an important mode of antioxidative mechanism (Ruberto et al. 2001). Moreover, most phenolic compounds found in the cyanobacteria extract contained the protonated form with the high chelating ability (Goh et al. 2010). The ability to chelate off the ferrous ion is important to avoid a Fenton reaction, which would produce the harmful free radicals such as O_2_^·−^. The presence of phenolic acids and flavonoids were supposed to impart prominent antioxidant properties that detoxify ROS products (Perron and Brumaghim 2009; Reuter et al. 2010; Singh et al. 2017).

Parallel action of non-enzymatic antioxidants, plant cells can scavenge toxicity of ROS products via enzymes such as superoxide dismutase (SOD), catalase (CAT), and other enzymatic antioxidants (Maffei et al. 2007; Ahmad et al. 2008). Potential roles of SOD and CAT in signaling and control of ROS production have been implicated in plant defense response to insect herbivores, including aphids (Ni et al. 2001; Gomez et al. 2004; Heng-Moss et al. 2004; Wei et al. 2007; Moloi and van der Westhuizen 2008; Rangasamy et al. 2009; Ferry et al. 2011; Mai et al. 2013). In agreement with the above results, our study revealed that the accumulated activity of SOD and CAT under infestation of *A. craccivora* is an aspect of antioxidative responses of *G. max* cv. “Nam Dan” to cowpea aphid. Within living cells, SOD constitutes the first line of defense against ROS (Alscher et al. 2002), because this enzyme can counteract oxidative damage caused by regulating levels of O_2_^·−^ (Abassi et al. 1998). The strong accumulated activity of CAT protects the plant cells against an excess of H_2_O_2_ and thus against considerable membrane damage (Rani and Jyothsna 2010). The potential defensive roles of SOD, CAT to control of ROS products have been involved in plant resistance to aphids (Rangasamy et al. 2009; Ferry et al. 2011).

Up to date, limited information has been mentioned about effects of cyanobacteria species on expression of the enzymatic antioxidants in plant defense mechanism. Inoculation cyanobacterial crude extract on alfalfa enhanced activities of SOD, CAT and other enzymes in response of alfalfa to oxidative stress (Pflugmacher et al. 2006). CAT activity significantly increased in *M. pigra* plant since 24 h after treating by *Nostoc* sp. extract (Sukkhaeng et al. 2015). Extract of *N. commune*, *Anabaena flos*-*acquae* and *Westiellopsis* sp. was found to enhance activity of CAT and POX of medicinal plant flax (Naresh et al. 2013). In soybean-cowpea aphid interaction, inoculation the aphid-infested leaves with *N. calcicola* HN9 extract improved activity of SOD and CAT (Figs. 7, 8). Because most cyanobacterial species contain a wide range of enzymes such as SOD, CAT, and other enzymatic antioxidants (Bharanidharan et al. 2013; Guedes et al. 2013; Gunes et al. 2015; Ghalab et al. 2016), SOD and CAT from *N. calcicola* HN9 extract probably augmented to the antioxidant defense system of soybean. The accumulated activity of SOD and CAT in soybean defense mechanism might be resulted from the acted combination or resonance of those enzymes originated from both soybean plant and cyanobacteria extract. The presence of high activity of enzymatic antioxidants in cyanobacteria extract may also be considered as a part of the key functions which contribute to protect of plants from effect of unfavorable factors, including aphids (Aydas et al. 2013; Singh et al. 2014).

Another important enzyme in H_2_O_2_-scavenging is GPx from the non-heme containing peroxidase family (Bela et al. 2015). Different from SOD and CAT, GPx was not detected in cyanobacteria (Tel-Or et al. 1986); therefore, the accumulated activity of this enzyme recorded in our study was originated only from soybean plant. An accumulation of GPx was as an aspect of soybean defense mechanism. GPx can regulate diverse defensive signaling pathways during oxidative stress and other unfavorable factors (Milla et al. 2003; Navrot et al. 2006; Diao et al. 2014; Gao et al. 2014). Typically, GPx is part of an alternative pathway that scavenges peroxides such as H_2_O_2_, phospholipid hydroperoxides, thereby protects cell membranes from peroxidative damage (Gueta-Dahan et al. 1997). It should be stressed that, inoculation of *N. calcicola* HN9 extract strongly improved activity of GPx in soybean leaves during cowpea aphid attack. This enzyme was accumulated since 6 hpi, reached to maximum activity at 48 hpi and maintained in high level up to ending of experiment; GPx activity was always significantly higher than that in soybean without inoculating cyanobacteria extract (Fig. 9).

Taken together, the aforementioned results provided the persuasive evidence that components of *N. calcicola* HN9 extract may strengthen the defensive capability of *G. max* cv. “Nam Dan” to cowpea aphid infestation via establishing the chemical constraints on oxidative stress. Cyanobacteria extract probably not only directly controlled the generation of O_2_^·−^ and H_2_O_2_ but also developed a range of chemical properties to limit aphid feeding. Furthermore, *N. calcicola* HN9 extract might augment activity of SOD and CAT, enhance action of GPx, allowed those enzymatic antioxidants to play crucial functions in defense mechanism of soybean “Nam Dan” to strictly reduce the toxic effects of ROS products.

Additionally, a high generation of O_2_^·−^ and H_2_O_2_, an accumulated activity of enzymatic antioxidants, and low cellular damages in leaves of *G. max* cv. “Nam Dan” after *A. craccivora* infestation suggest that it may be a aphid resistant variety of soybean.

In conclusion, the enzymatic antioxidants such as SOD, CAT and GPx are important elements of soybean defense responses against cowpea aphid infestation. The accumulation of those enzymes can be thought of a reflection of intrinsic strategies to reduce damages caused due to ROS generated during aphid attack. Inoculation soybean plant with *N. calcicola* HN9 extract, which contains various enzymatic and non-enzymatic antioxidants, strengthened the adaptive potential to protect soybean from oxidative stress, contributed to increase the resistance of *G. max* cv. “Nam Dan” to *A. craccivora*. The improved antioxidative responses of *G. max* cv. “Nam Dan” to *A. craccivora* infestation under effect of *N. calcicola* HN9 extract can be a novel aspect of bio-protection, which is an integral component of the friendly strategies for sustainable agriculture.

## Abbreviations

CAT: catalase
FW: fresh weight
GPx: glutathione peroxidase
H_2_O_2_: hydrogen peroxide
hpi: hours post infestation
nkat: nanokatal
O_2_^·−^: superoxide anion radical
SOD: superoxide dismutase
